# Supplementary material for: Determinants of health as predictors for differential antibody responses following SARS-CoV-2 primary and booster vaccination in an at-risk, longitudinal cohort
Source: PLoS One. 2024 Apr 2;19(4):e0292566. doi: 10.1371/journal.pone.0292566 (PMC10987003; doi:10.1371/journal.pone.0292566)
Supplement: S1 Table — (PDF) [file pone.0292566.s001.pdf]

S1 Table. Other Measured Extrinsic and Intrinsic Health Factors Among the Primary Vaccination Sub-Cohort.

PRIMARY VACCINATION			
EXTRINSIC HEALTH FACTORS	N	Mean $\log_2$ Ab (± SD)	*p*
Alcohol Consumption *(n = 148)*			
No	29 (19.59%)	12.75 (± 1.97)	0.063
Yes	119 (80.41%)	13.39 (± 1.58)	
Cardiovascular Disease *(n = 146)*			
No	141 (96.58%)	13.29 (± 1.59)	0.555
Yes	5 (3.42%)	12.84 (± 3.11)	
COVID-19 Vaccine Manufacturer *(n = 149)*			
Moderna mRNA-1273	54 (36.24%)	13.50 (± 1.71)	0.211
Pfizer BNT162b2	95 (63.76%)	13.14 (± 1.64)	
Diabetes *(n = 147)*			
No	143 (97.28%)	13.31 (± 1.61)	0.619
Yes	4 (2.72%)	12.89 (± 2.87)	
Hypercholesterolemia *(n = 146)*			
No	112 (80.58%)	13.31 (± 1.60)	0.405
Yes	27 (19.42%)	13.01 (± 1.96)	
Hypertension *(n = 146)*			
No	124 (84.93%)	13.34 (± 1.50)[a]	0.225
Yes	22 (15.07%)	12.69 (± 2.36)	
INTRINSIC HEALTH FACTORS	N	Mean $\log_2$ Ab (± SD)	*p*
Education Level *(n = 149)*			
Associate degree or technical degree	12 (8.05%)	13.56 (± 1.44)	0.120
Bachelor's degree	30 (20.13%)	13.68 (± 1.69)	
High school diploma or equivalent	9 (6.04%)	13.98 (± 1.58)	
Master's degree or higher	75 (50.34%)	13.16 (± 1.64)	
Other	23 (15.44%)	12.64 (± 1.76)	
Marital Status *(n = 149)*			
Divorced	12 (8.05%)	13.81 (± 1.24)	0.257
Domestic partnership	7 (4.70%)	13.07 (± 1.51)	
Married	81 (54.36%)	13.04 (± 1.82)	
Single	47 (31.54%)	13.54 (± 1.49)	
Widowed	**2** (1.35%)	13.64 (± 0.00)	
Race *(n = 149)*			
Asian	9 (6.04%)	13.09 (± 1.88)	0.712
Black	6 (4.03%)	13.98 (± 0.82)	
Other	14 (9.40%)	13.43 (± 1.37)	
White	120 (80.54%)	13.23 (± 1.72)	
Sexual Orientation *(n = 146)*			
Bisexual	**2** (1.34%)	14.14 (± 0.71)	0.853
Gay	10 (6.71%)	13.34 (± 0.45)	
Heterosexual	134 (89.94%)	13.24 (± 0.15)	
Lesbian	**2** (1.34%)	12.64 (± 0.00)	
Other	1 (0.67%)		

[a]Levene's test found a statistically significant difference in the variances between participants with and without hypertension ($F(1,145) = 8.15$, $p = 0.005$).
