# Supplementary material for: Determinants of health as predictors for differential antibody responses following SARS-CoV-2 primary and booster vaccination in an at-risk, longitudinal cohort
Source: PLoS One. 2024 Apr 2;19(4):e0292566. doi: 10.1371/journal.pone.0292566 (PMC10987003; doi:10.1371/journal.pone.0292566)
Supplement: S2 Table — (PDF) [file pone.0292566.s002.pdf]

S2 Table. Linear Mixed Effects Model (LMM) Evaluating the Relationship Between Post-Primary Vaccination Antibody Titers and Time, COVID-19 Vaccine Manufacturer, Prior COVID-19 Infection Status, and Biological Sex.

**(Intercept)**	**numDF**	**denDF**	**F-value**	**p-value**
(Intercept)	1	524	19567.841	0.000
daysSinceFullVax	1	524	96.889	**0.000***
daysSinceFullVax2	1	524	118.393	**0.000***
daysSinceFullVax3	1	524	44.654	**0.000***
CovidVaxManufacturer	1	108	36.900	**0.000***
CovidStatus	1	108	64.917	**0.000***
Gender	1	108	4.856	**0.030***
ageAtEntry	1	108	1.793	0.183
DrugUse	1	108	1.477	0.227
Ethnicity	1	108	0.071	0.790
Race	3	108	0.087	0.967
