# Supplementary material for: Determinants of health as predictors for differential antibody responses following SARS-CoV-2 primary and booster vaccination in an at-risk, longitudinal cohort
Source: PLoS One. 2024 Apr 2;19(4):e0292566. doi: 10.1371/journal.pone.0292566 (PMC10987003; doi:10.1371/journal.pone.0292566)
Supplement: S3 Table — (PDF) [file pone.0292566.s003.pdf]

S3 Table. Other Measured Extrinsic and Intrinsic Health Factors Among the First Booster Vaccination Sub-Cohort.

BOOSTER VACCINATION I			
EXTRINSIC HEALTH FACTORS	N	Mean log_2 Ab (± SD)	*p*
Alcohol Consumption *(n = 97)*			
No	23 (23.71%)	14.43 (± 1.48)	0.124
Yes	74 (76.29%)	14.85 (± 1.01)	
Cardiovascular Disease *(n = 95)*			
No	92 (96.84%)	14.75 (± 1.15)	0.872
Yes	3 (3.16%)	14.64 (± 1.00)	
COVID-19 Vaccine Manufacturer *(n = 97)*			
Moderna mRNA-1273 – Full	17 (17.52%)	14.82 (± 1.55)	0.087
Moderna mRNA-1273 – Half	24 (24.74%)	15.14 (± 0.78)	
Pfizer BNT162b2	56 (56.74%)	14.54 (± 1.10)	
Diabetes *(n = 96)*			
No	93 (96.88%)	14.77 (± 1.14)	0.239
Yes	3 (3.12%)	13.98 (± 1.53)	
Hypercholesterolemia *(n = 95)*			
No	66 (74.16%)	14.81 (± 1.25)	0.781
Yes	23 (25.84%)	14.73 (± 0.95)	
Hypertension *(n = 95)*			
No	77 (81.05%)	14.75 (± 1.12)	0.836
Yes	18 (18.95%)	14.81 (± 0.22)	
INTRINSIC HEALTH FACTORS	N	Mean log_2 Ab (± SD)	*p*
Education Level *(n = 98)*			
Associate degree or technical degree	10 (10.20%)	14.84 (± 1.62)	0.916
Bachelor's degree	21 (21.43%)	14.64 (± 1.38)	
High school diploma or equivalent	**2** (2.04%)	14.14 (± 0.71)	
Master's degree or higher	48 (48.98%)	14.81 (± 0.91)	
Other	17 (17.35%)	14.64 (± 1.23)	
Marital Status *(n = 98)*			
Divorced	8 (8.16%)	15.02 (± 1.06)	0.869
Domestic partnership	4 (4.08%)	14.39 (± 0.96)	
Married	61 (62.24%)	14.68 (± 1.22)	
Single	22 (22.45%)	14.83 (± 1.01)	
Widowed	3 (3.07%)	14.98 (± 1.16)	
Race *(n = 98)*			
Asian	8 (8.16%)	14.77 (± 0.84)	0.747
Black	3 (3.06%)	15.32 (± 0.58)	
Other	5 (5.11%)	15.04 (± 0.55)	
White	82 (83.67%)	14.69 (± 1.21)	
Sexual Orientation *(n = 95)*			
Bisexual	**1** (1.02%)	--	0.368
Gay	6 (6.12%)	14.31 (± 0.82)	
Heterosexual	90 (91.84%)	14.74 (± 1.15)	
Lesbian	**1** (1.02%)	--	
Other	**0** (0.00%)	--	
