# Supplementary material for: Determinants of health as predictors for differential antibody responses following SARS-CoV-2 primary and booster vaccination in an at-risk, longitudinal cohort
Source: PLoS One. 2024 Apr 2;19(4):e0292566. doi: 10.1371/journal.pone.0292566 (PMC10987003; doi:10.1371/journal.pone.0292566)
Supplement: S4 Table — (PDF) [file pone.0292566.s004.pdf]

**S4 Table. Linear Mixed Effects Model (LMM) Evaluating the Relationship Between BV1 Antibody Titers and Time, COVID-19 Vaccine Manufacturer, Prior COVID-19 Infection Status, and Biological Sex.**

| | **numDF** | **denDF** | **F-value** | **p-value** |
|---|---|---|---|---|
| *(Intercept)* | 1 | 524 | 20272.867 | <.0001 |
| daysSinceBoost1 | 1 | 72 | 0.012 | 0.914 |
| daysSinceBoost1^2 | 1 | 72 | 4.007 | **0.049*** |
| daysSinceBoost1^3 | 1 | 72 | 10.142 | **0.002*** |
| ageAtEntry | 1 | 24 | 0.290 | 0.595 |
| BoosterType | 2 | 24 | 4.775 | **0.018*** |
| Gender | 1 | 24 | 0.667 | 0.422 |
| Race | 2 | 24 | 0.123 | 0.885 |
| Ethnicity | 1 | 24 | 1.179 | 0.288 |
| DrugUse | 1 | 24 | 8.917 | **0.006*** |
