# Supplementary material for: Determinants of health as predictors for differential antibody responses following SARS-CoV-2 primary and booster vaccination in an at-risk, longitudinal cohort
Source: PLoS One. 2024 Apr 2;19(4):e0292566. doi: 10.1371/journal.pone.0292566 (PMC10987003; doi:10.1371/journal.pone.0292566)
Supplement: S5 Table — (PDF) [file pone.0292566.s005.pdf]

S5 Table. Other Measured Extrinsic and Intrinsic Health Factors Among the Second Booster Vaccination Sub-Cohort.

BOOSTER VACCINATION II			
EXTRINSIC HEALTH FACTORS	N	Mean log_2 Ab (± SD)	*p*
Alcohol Consumption *(n = 23)*			
No	6 (26.09%)	14.43 (± 1.48)	0.124
Yes	17 (73.91%)	14.85 (± 1.01)	
Cardiovascular Disease *(n = 22)*			
No	20 (90.91%)	15.19 (± 1.70)	--
Yes	**2** (9.09%)	14.64 (± 1.41)	
COVID-19 Vaccine Manufacturer *(n = 24)*			
Moderna mRNA-1273 – Full	13 (54.17%)	15.41 (± 1.74)	0.253
Pfizer BNT162b2	11 (45.83%)	14.64 (± 1.41)	
Diabetes *(n = 23)*			
No	21 (91.30%)	13.03 (± 1.66)	--
Yes	**2** (8.70%)	16.14 (± 0.71)	
Hypercholesterolemia *(n = 23)*			
No	11 (52.38%)	14.92 (± 2.01)	0.383
Yes	10 (47.62%)	15.54 (± 0.99)	
Hypertension *(n = 22)*			
No	20 (90.91%)	15.19 (± 1.70)	--
Yes	**2** (9.09%)	14.64 (± 1.41)	
INTRINSIC HEALTH FACTORS	N	Mean log_2 Ab (± SD)	*p*
Education Level *(n = 24)*			
Associate degree or technical degree	**2** (8.33%)	15.64 (± 2.83)	0.556
Bachelor's degree	5 (20.83%)	14.44 (± 2.39)	
High school diploma or equivalent	--	--	
Master's degree or higher	13 (54.17%)	14.03 (± 1.26)	
Other	4 (16.67%)	15.64 (± 1.41)	
Marital Status *(n = 24)*			
Divorced	3 (12.50%)	15.98 (± 0.58)	0.261
Domestic partnership	**1** (4.17%)	--	
Married	16 (66.67%)	14.77 (± 1.75)	
Single	**2** (8.33%)	15.14 (± 0.71)	
Widowed	**2** (8.33%)	14.64 (± 1.41)	
Race *(n = 24)*			
Asian	**2** (8.33%)	14.14 (± 2.12)	--
Black	**2** (8.33%)	15.64 (± 0.00)	
Other	**2** (8.33%)	15.14 (± 0.71)	
White	18 (75.01%)	15.09 (± 1.76)	
Sexual Orientation *(n = 22)*			
Bisexual	**0** (0.00%)	--	--
Gay	**2** (8.33%)	15.64 (± 2.83)	
Heterosexual	22 (91.67%)	15.01 (± 1.56)	
Lesbian	**0** (0.00%)	--	
Other	**0** (0.00%)	--	
