# Supplementary material for: Determinants of health as predictors for differential antibody responses following SARS-CoV-2 primary and booster vaccination in an at-risk, longitudinal cohort
Source: PLoS One. 2024 Apr 2;19(4):e0292566. doi: 10.1371/journal.pone.0292566 (PMC10987003; doi:10.1371/journal.pone.0292566)
Supplement: S6 Table — (PDF) [file pone.0292566.s006.pdf]

S6 Table. Linear Mixed Effects Model (LMM) Evaluating the Relationship Between BV2 Antibody Titers and Time, COVID-19 Vaccine Manufacturer, Prior COVID-19 Infection Status, and Biological Sex.

	**numDF**	**denDF**	**F-value**	**p-value**
(Intercept)	1	104	4658.924	<.0001
daysSinceBoost2	1	104	1.468	0.228
daysSinceBoost2^2	1	104	7.916	**0.006***
daysSinceBoost2^3	1	104	0.114	0.737
ageAtEntry	1	23	0.058	0.811
Booster2Type	2	23	2.779	0.083
CovidStatusNegative	1	23	0.475	0.498
GenderMale	1	23	3.496	0.074
Race	2	23	0.221	0.803
Ethnicity	1	23	0.151	0.701
DrugUse	1	23	3.208	0.087
